# Comparative Study of the Tensile Properties of a Zircaloy-4 Alloy Characterized by Mesoscale and Standard Specimens

**DOI:** 10.3390/ma18030666

**Published:** 2025-02-03

**Authors:** Ruohan Dong, Ning Zhao, Shenghui Tong, Zeen Zhang, Gang Li, Zesheng You

**Affiliations:** 1 Herbert Gleiter Institute of Nanoscience, School of Materials Science and Engineering, Nanjing University of Science Technology, Nanjing 210094, China; 123116011395@njust.edu.cn (R.D.); zhaoning123@njust.edu.cn (N.Z.); shenghuitong@njust.edu.cn (S.T.); zeenzhang@njust.edu.cn (Z.Z.); 2 National Key Laboratory of Nuclear Reactor Technology, Nuclear Power Institute of China, Chengdu 610041, China

**Keywords:** mechanical tests, size effect, zirconium alloy, femtosecond laser, small scale, mesoscale

## Abstract

The accuracy and reliability of small-scale mechanical tests remain doubtful due to significant dependence of the obtained mechanical properties on specimen size. Mesoscale tensile tests with specimen sizes ranging from 10 μm to 1 mm are capable of obtaining bulk-like properties but are rarely applied to hexagonal close-packed metals. In this study, well-designed comparative tensile tests were carried out on a Zircaloy-4 alloy with a grain size of 4 μm using femtosecond laser-machined mesoscale specimens with a thickness of about 60 μm, sub-sized specimens with a thickness of about 1.3 mm, and standard specimens with a thickness of 4 mm. The quantitative results revealed that irrespective of the small specimen dimensions, the yield strength, tensile strength, and tensile ductility are only approximately 10.4%, 5.2%, and 13% lower than those of the standard specimens, respectively. This clearly demonstrates that the mechanical properties can be assessed with satisfactory accuracy by mesoscale tensile tests. The comparatively greater deviation of the yield strength at the mesoscale arises from the disappearance of yield point behavior, while the reduced tensile ductility is associated with the larger volume fraction of surface grains. The surface grains are characterized by more surface dislocation sources and deform with weaker constraints from neighboring grains, leading to smooth plastic yielding and slightly reduced strain hardening at the mesoscale.

## 1. Introduction

Evaluation of mechanical properties at small length scales has continuously attracted extensive attention all over the world [1,2,3,4,5,6]. Such small-scale mechanical tests are frequently unavoidable once the materials available for testing are scarce or expensive. These scenarios include materials after a long duration of neutron or ion irradiation [2,7,8], novel nanostructured materials produced by electro-deposition or severe plastic deformation [9,10,11,12,13], and samples extracted from in-service components for monitoring property degradation [14]. Meanwhile, miniaturized mechanical testing is also advantageous for probing properties of specific microstructural features of interest, such as single grains, grain boundaries, interfaces, and heat-affected zones of weldments [15,16,17,18,19].

Small-scale mechanical tests generally fall into two categories at different scales. The first category utilizes sub-sized samples with minimum dimensions in the order of millimeters [20,21]. Mechanical tests at this length scale can be easily conducted with either standard or miniaturized testers [22]; however, the sample sizes are still too large when the sample volume is extremely limited. The second category employs microscale or even nanoscale specimens that are commonly fabricated by focused ion beam (FIB) milling and tested in situ within an electron microscope [23,24,25]. Due to the extremely low milling rates and high costs, FIB milling can only produce specimens with dimensions smaller than 10 μm for reasonable periods of milling time [26]. As a consequence, only single-crystal or bi-crystal samples can be tested, and the results exhibit a significant sample size effect and distinctly deviate from those of bulk materials [4,27]. Evidently, there is an intermediate gap ranging from 10 μm to 1 mm that remains largely unexplored. The tests within this length scale gap belong to the so-called mesoscale mechanical tests [2,28,29].

Tensile tests at the mesoscale have several advantages. Firstly, the samples can incorporate sufficient microstructural constituents to acquire mechanical properties comparable to those obtained by bulk counterparts [20]. For instance, it is well established that the ratio of minimum sample dimension (thickness or diameter) to grain size should be greater than a critical value to acquire size-independent flow strengths. For most engineering materials with grain sizes in the micrometer scale, the minimum sample dimension should range from tens to hundreds of micrometers, just falling into the mesoscale range. Secondly, the tensile tests can provide a wealth of information, including not only strengths but also strain hardening rate and plasticity. Thirdly, the tests at mesoscale can be carried out in situ under an optical microscope instead of within an expensive electron microscope and thus require much lower investment on the testing instrument [30,31].

The limited mesoscale tensile tests are primarily caused by a lack of efficient sample preparation tools. The sample size at the mesoscale is too large for FIB milling yet too small for convention techniques [26]. In recent years, femtosecond laser micro-machining has gradually emerged as a viable technology for efficiently producing specimens for mesoscale mechanical characterizations [32,33,34,35]. Since the laser pulse width (typically <500 fs) is shorter than the time for thermal diffusion, the femtosecond laser ablation becomes athermal, and the heat-affected regions are limited to low degrees [36,37]. However, the materials may still experience dislocation injection, phase transformation, or recrystallization if ablated at high-energy fluences [38,39,40,41]. The potential damages caused by femtosecond laser can approach a maximum depth of several micrometers. Such laser damage layers can be removed by FIB milling [42] or by electro-chemically polishing [30]. Alternatively, it has also been demonstrated by Bao et al. that the surface damage layer can be directly cleared by conducting sidewise laser polishing [31]. So far, femtosecond laser machining has been employed to prepare samples of various materials for small-scale mechanical testing, such as lamellar structured stainless steel [30], copper [31], nano-grained Ni [43], nanovoids-dispersed Au [44], high-entropy alloys [13], and bulk metallic glass [45]. Most of these materials have a face-centered cubic crystal structure. There are still limited mesoscale mechanical tests on hexagonal close-packed (HCP) metals.

Moreover, the accuracy and reliability of the mechanical properties derived from mesoscale specimens prepared by femtosecond laser ablation still remain doubtful. The mechanical properties, in particular the tensile ductility, depend strongly on sample size, sample geometry, and microstructural length scale [20,46]. The accuracy of the results is also influenced by the quality of sample preparation and the reliability of the testing instruments, especially the accuracy of strain measurement. In order to verify the fidelity of the results, it is highly necessary to conduct systematical comparative studies between mesoscale and standard specimens of various materials. However, to the best of our knowledge, there is no report on such comparative investigations.

Taking a zirconium alloy as an example of HCP metals, this work aims to perform well-designed comparative tensile tests of standard specimens on commercial mechanical testing instruments and of femtosecond laser-machined mesoscale specimens on a custom-developed small-scale mechanical testing platform. All the parameters that might affect the accuracy of results, such as quality of machined specimen, sample geometry, accuracy of strain measurement, and precision of testing apparatus, are optimized to keep their influences to minimal levels. The results clearly demonstrate that the mesoscale tensile tests can obtain mechanical properties that are in satisfactory agreement with those of standard specimens. Our findings highlight the feasibility of measuring bulk mechanical properties via miniaturized specimens at the mesoscale once some critical experimental conditions are fulfilled.

## 2. Materials and Methods

### 2.1. Material

The material investigated in this study is a commercial Zircaloy-4 alloy. The chemical composition of the alloy is given in Table 1. The sample was supplied in the form of rolled and recrystallized plate with an initial thickness of about 4 mm.

### 2.2. Femtosecond Laser Micromachining

**Figure 1:**
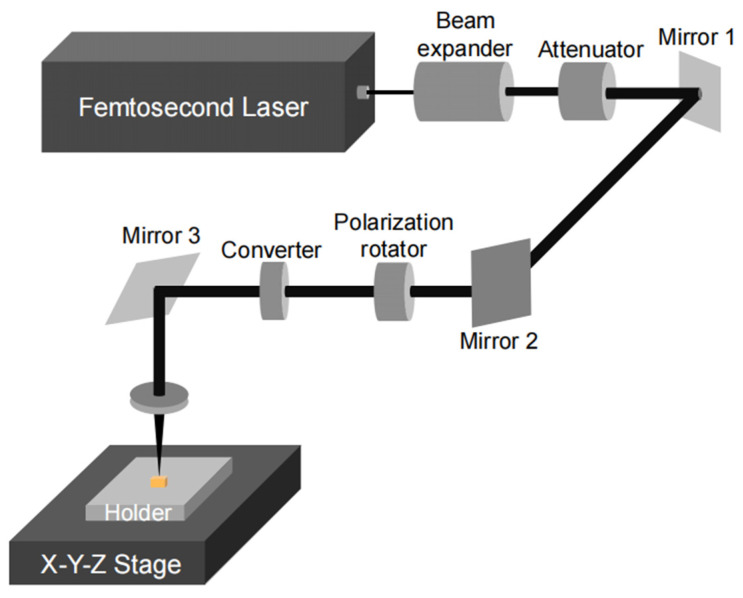
Schematic of the laser beam path in the fs-laser micromachining system.

Femtosecond laser micromachining was employed to machine the mesoscale tensile specimens. The laser machining was performed on a commercial femtosecond laser micromachining system (FemtoFAB, Workshop of Photonics, Vilnius, Lithuania). The wavelength of the laser beam is 343 nm, and the pulse width is 280 fs. As shown in Figure 1, the laser beam passes through a beam expander, a motorized laser power attenuator, a motorized polarization rotator, a linear-to-circular polarization converter, and is finally focused by an objective lens to a spot with a radius of ~5.0 μm on the sample surface. During the machining, the laser beam is kept fixed and perpendicular to the sample plane, while the sample is moved by a three-axis Aerotech motion stage with a positional accuracy of ±300 nm at a speed of 1 mm s^−1^ to repeat scanning of the cutting path for more than 60 times. The position synchronized output (PSO) feature is supported in the laser system, which generates uniform laser pulses along the machining trajectory. In this study, the pulse density was set to 5000 pulses mm^−1^, which corresponds to about 98% of spot overlap.

### 2.3. Tensile Tests

**Figure 2:**
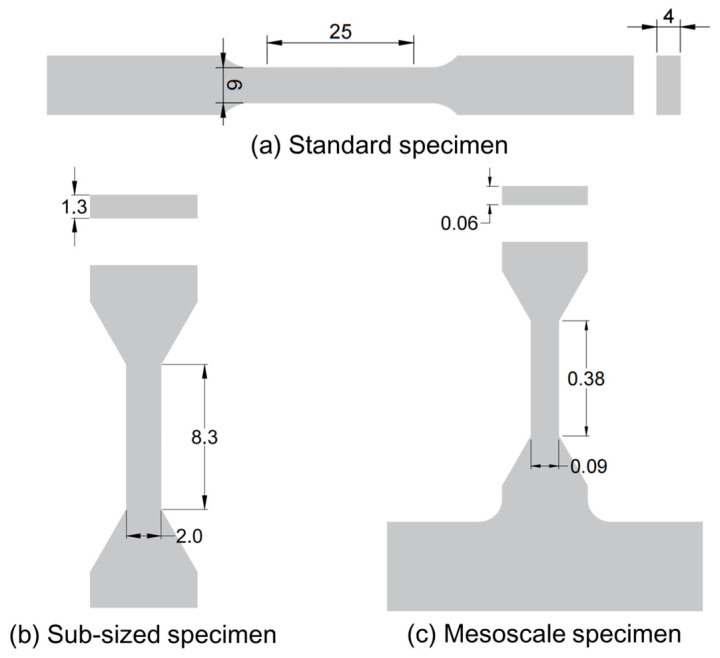
Schematic showing the dimensions of (**a**) standard, (**b**) sub-sized, and (**c**) mesoscale tensile specimen in units of mm.

The mechanical properties of the Zircaloy-4 alloy were evaluated with dog-bone-shaped tensile specimens at three different length scales, as schematically shown in Figure 2. The one at the largest scale, hereinafter referred to as the standard sample, is designed strictly according to the ASTM E8/E8M-22 standard [47] and has a gauge length (*L*) of 25 mm, a gauge width (*w*) of 6 mm, and a thickness (*t*) of 4 mm. The one at the medium scale, hereinafter referred to as sub-sized sample, has a gauge section with *L* = 8.3 mm, *w* = 2 mm, and *t* = 1.3 mm. The one at the smallest scale, hereinafter referred to as mesoscale sample, has a gauge section with *L* = 380 μm, *w* = 90 μm, and *t* = 60 μm. The sub-sized and mesoscale samples were proportionally scaled down from the standard sample in order to eliminate the influence of sample geometry on the mechanical properties.

All the samples for the tensile tests were extracted from the same Zircolay-4 plate along the same direction. Both the standard and sub-sized specimens were cut by electro-discharging machine and then mechanically ground to the required thickness. For the mesoscale tensile tests, thin sheets were cut from the Zircolay-4 plate and then mechanically ground and polished to a final thickness of about 60 μm. For ease of handling, the thin sheets were glued to a 1 mm thick copper plate using cyanoacrylate adhesive prior to the laser machining. Then, the mesoscale specimens were cut from the thin sheets by the femtosecond laser micromachining. After the samples were cut out, additional sidewise polishing by laser beam was conducted to increase the cut surface quality, as described in detail in Ref. [31]. The real dimensions of the mesoscale tensile specimens were measured by an optic microscope, and the accuracy of dimension measurement is about ±2 μm.

**Figure 3:**
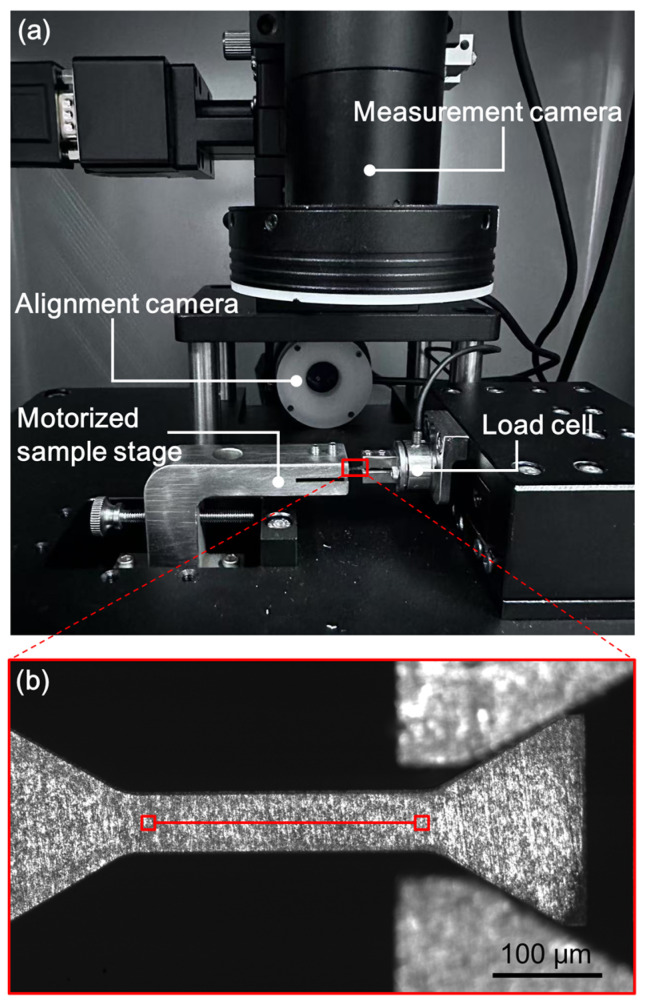
(**a**) Photo of the custom-developed mesoscale mechanical testing instrument. (**b**) A captured digital image showing a tensile specimen that has been installed into the tungsten gripper. The two squares in (**b**) indicate the two 41 × 41 subsets of pixels manually picked to represent the gauge.

Owing to the large differences in the sample dimensions, the tensile tests were conducted in different machines with different load capacities. The standard samples were tested on a universal testing machine (Instron 5982, Norwood, MA, USA) equipped with a load cell of 100 kN, and the sub-sized specimens were tested on a universal testing machine (Instron 5982, Norwood, MA, USA) equipped with a load cell of 5 kN. The strain of the standard and sub-sized specimens was measured by a contactless video extensometer based on digital image correlation (DIC), as described in detail elsewhere [22,48]. The mesoscale tensile tests were carried out on a custom-developed small-scale mechanical testing instrument, as shown in Figure 3a. The testing instrument consists of a piezo-ceramic driving motor, a high-precision miniaturized load cell with a capacity of 10 N, a motorized sample stage, a side-view alignment camera, and a top-view measurement camera. The load cell was calibrated by standard known weights and had a resolution of 10 mN. The measurement camera with a telecentric lens of 10× magnification was used to capture digital images of the sample surface for computing the elongation of the gauge by DIC. The mesoscale sample fixed on the Cu plate was installed into the sample stage and moved into a tungsten gripper installed on the load cell. Prior to the testing, an image was captured for reference image, as shown in Figure 3b, and two 41 × 41 subsets of pixels were manually picked on both ends of the gauge to represent gauge length. All the tensile tests were conducted under displacement control, with displacement rates of 1.5, 0.5, and 0.023 mm min^−1^ for standard, sub-sized, and mesoscale specimens, respectively, corresponding to a constant nominal strain rate of about 1 × 10^−3^ s^−1^ at different length scales. To guarantee reproducibility, the tensile tests were repeated more than three times.

### 2.4. Microstructural Characterization

To characterize the material microstructure, specimens were mechanically polished with progressively finer silicon carbide papers and abrasive slurries. In order to further remove the surface damage layer, the specimens were additionally treated by vibratory polishing with 50 nm colloidal silica suspension for 12 h. The specimens were then heat tinted at 370 °C for 5 min and observed by polarized light optical microscope. The roughness of the cut surface was analyzed by a confocal laser scanning microscope (CLSM, Olympus LEXT OLS4100, Tokyo, Japan). The fracture surface morphologies were examined by scanning electron microscope (SEM, CIQTEK 3200A, Hefei, China) at an accelerating voltage of 15 kV.

## 3. Results

### 3.1. Microstructure

**Figure 4:**
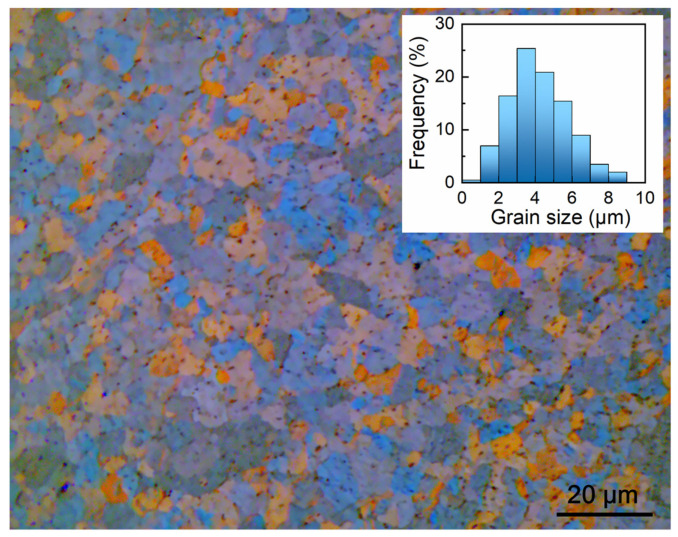
Polarized light optical microscopy image showing the grain structure of the Zircaloy-4 alloy. The inset shows the statistical histogram of grain size.

Figure 4 displays the polarized light microscope observations of the grain structure of the Zircaloy-4 alloy. The microstructure is homogenous with equiaxed grains. The inset of Figure 4 shows the statistical histogram of measured grain sizes. The grain size (*d*) falls in the ranges of 1 to 10 µm, with an average value of 4.0 µm. Therefore, even for the mesoscale tensile tests, the ratio of sample thickness to grain size (*t/d*) is about 15. Such a comparatively high *t/d* ratio is important for obtaining bulk mechanical properties with small-scale mechanical tests.

### 3.2. Cut Surface Quality

**Figure 5:**
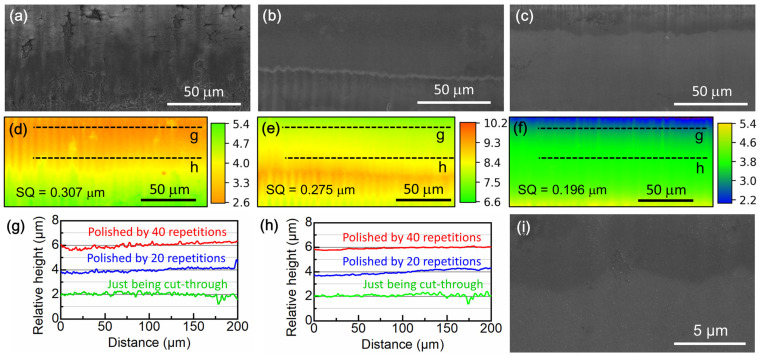
(**a**–**c**) SEM images of the laser-machined surface of Zircaloy-4 alloy subjected to different repetitions of scanning during the sidewise polishing stage: (**a**) just being cut through; (**b**) 20 repetitions of scanning; (**c**) 40 repetitions of scanning. (**d**–**f**) Corresponding CLSM relative height pseudo-color maps of (**a**–**c**), respectively. (**g**,**h**) Surface relative height profiles on the top edge (**g**) and in the middle (**h**) of the machined surface sidewise polished by different repetitions of scanning. (**i**) High-magnification SEM image of the area in the middle of the machined surface in (**c**). The laser incidence direction is from top to bottom.

The femtosecond laser machining of tensile specimens from thin metal sheets by direct writing method consists of two stages: the cut-through stage and the sidewise polishing stage [31]. During the cut-through stage, the laser beam is perpendicular to the sample surface, and the material below the cutting path is ablated at very high laser fluences in the center peak of the Gaussian beam. As a consequence, melting might take place for most materials, and high dislocation densities or amorphization may develop beneath the cut surface [38]. Figure 5a displays the machined surface when the thin sheet is just cut through. The surface is fairly rough, with obvious cutting traces parallel to the laser incidence direction. Moreover, most of the surface appears to be covered by a layer of ablation debris.

In order to reduce the influence of laser damage on mechanical properties, an additional sidewise polishing stage should be conducted to enhance the quality of the cut surface. During the sidewise polishing stage, the laser cutting trajectory is scanned for an additional 40 repetitions, with the trajectory contracted 1 μm inward after every 5 repetitions of scanning. Since the laser beam is almost parallel to the cut surface in this stage, the surface is ablated by the rim of the Gaussian beam, where the laser fluence is just slightly greater than the laser ablation threshold [31]. Due to the low laser fluence, the surface damage layer produced in the cut-through stage is gradually removed while not introducing new laser damage. Figure 5b,c show the cut surface morphologies after being polished for 20 and 40 repetitions of scanning, respectively. Evidently, the sidewise polishing gradually removes the surface-deposited debris and laser-cutting traces. The cut surface becomes fairly smooth after 40 repetitions of scanning. Figure 5d–f display the corresponding CSLM relative height maps of the cut surface in Figure 5a–c, respectively. Owing to the cone shape of the focused laser beam, the cut surface has a taper angle of 3 to 5°, as shown by the color gradient. The computed surface roughness represented by root-mean-squared height (SQ) is marked in Figure 5d–f. The polishing reduces SQ from 0.307 to 0.197 μm. The relative height profiles on the top edge corners and in the middle of the cut surface are shown in Figure 5g,h, respectively. It is noted that the surface roughness on the top edge color is always greater than that in the center of the cut surface. Figure 5i presents the magnified observations of the area in the middle of the cut surface. The surface is very smooth, with only small stochastic dots. Previous characterization of the microstructure beneath the smooth cut surface reveals that the laser damages (dislocation injections) can only reach a depth smaller than 500 nm after the laser sidewise polishing and occupy a volume fraction of ~1.5%. Therefore, their influence on the mechanical properties is negligible [31].

### 3.3. Tensile Properties

**Figure 6:**
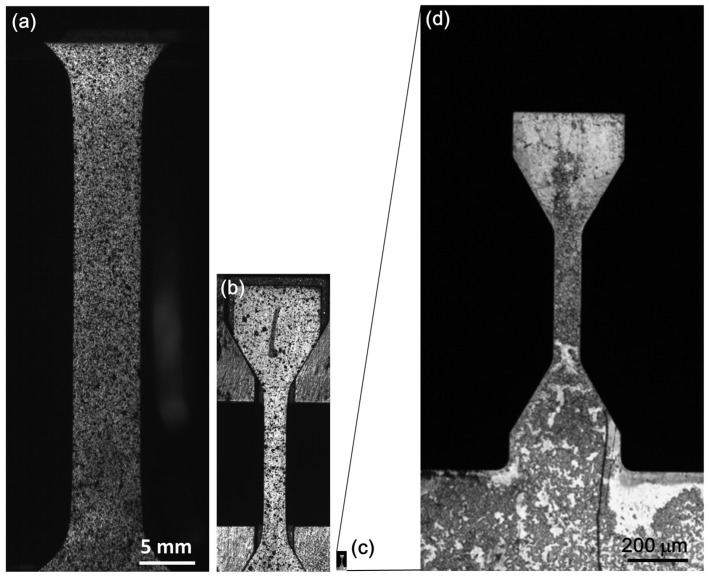
Captured digital images of the tensile specimens at different length scales: (**a**) Standard specimen; (**b**) sub-sized specimen; (**c**,**d**) mesoscale specimen.

Tensile tests of the zircolay-4 alloy were carried out at ambient temperature on three different scales. Figure 6a–c display the captured digital photos of the standard, sub-sized, and mesoscale dog-bone-shaped tensile specimens, respectively. Due to the extremely small dimension, the mesoscale specimen is also magnified, as shown in Figure 6d. The differences in the sample dimensions are obvious. In fact, the gauge section volume of the mesoscale specimen is only 3.38 ppm of that of the standard specimen. The random patterns were made on the sample surface to measure gauge extension using the DIC principle.

**Figure 7:**
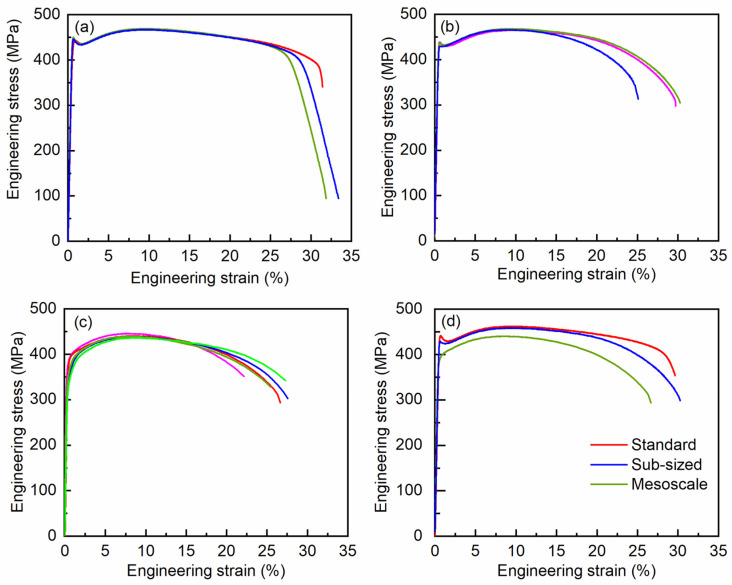
Engineering stress and engineering strain curves of the tensile tests at different length scales: (**a**) standard specimen; (**b**) sub-sized specimen; (**c**) mesoscale specimen. (**d**) Comparison of tensile curves at different length scales. The different colors of the curves in (**a**–**c**) represent results from repeated tensile tests.

In order to show the reproducibility of the results, Figure 7a–c show all the obtained engineering stress–engineering strain curves for the standard, sub-sized, and mesoscale specimens, respectively. Figure 7d presents the comparison of representative tensile curves at different length scales. The derived mechanical properties, including 0.2% offset yield strength (YS), ultimate tensile strength (UTS), uniform elongation (UE), and failure elongation (FE), are listed in Table 2. For each length scale, the tensile curves are mutually consistent with each other, an indication of the high precision of the results. The standard deviations (STDs) of YS and UTS (13 and 15 MPa, respectively) of the mesoscale specimens are greater than those of the standard and sub-sized specimens, suggesting a comparatively lower precision of the strength measurement. The lower precision at the mesoscale is probably associated with the relatively lower reliability of dimension measurement for such miniaturized specimens. As an estimate, the STDs of gauge width and thickness are about ±2 μm. Based on error propagation analysis, this contributes to a relative error of approximately 4% on the calculated stress, namely 16 and 18 MPa for YS and UTS, respectively, which are in agreement with the corresponding experimental values. Different from the stresses, UE and FE show analogous STD values at different length scales because they are measured by the same DIC gauging system.

In order to quantify how significantly the mechanical properties measured by small-size specimens differ from those by standard specimens, a Student’s *t*-test statistical analysis was conducted. Table 3 summarizes the corresponding t-statistic values and *p*-values of the mechanical properties measured by sub-sized and mesoscale specimens with respect to those by standard specimens. For sub-sized specimens, the *t*-test analysis shows the *p*-values of the mechanical properties are greater than the significance level (defined as 0.5). Therefore, the mechanical properties of the sub-sized tensile specimens are not significantly different from those of the standard specimens. On the contrary, the *p*-values for the mesoscale specimens are smaller than 0.5. This suggests that the mechanical properties obtained by mesoscale specimens deviate statistically significantly from those by standard specimens. Therefore, the accuracies of mechanical properties measured by small-size tensile tests are analyzed. The accuracies, defined as the relative deviations of the mechanical properties from those of standard specimens, are included in Table 3. The accuracies of YS and UTS of the mesoscale specimens are –10.4% and –5.2%, respectively. The UE and FE values are −12.5% and −13.5% lower than those of the standard specimens. The minor relative deviations show that the mechanical properties obtained by the mesoscale specimens are still satisfactorily consistent with those of the standard specimens.

Compared with UTS, the YS of the mesoscale specimens exhibits a comparatively greater deviation (−10.4%) from those of the standard specimens. Inspection of the tensile curves reveals that the lower YS values are intimately associated with the different yielding behavior at the macroscale and mesoscale. For the standard and sub-sized specimens, the tensile curves display an evident yield point behavior; namely, the flow strength slightly decreases immediately after the plastic yielding and then increases again with further tensile loading. Such a yield drop behavior in zirconium alloy has also been reported in the literature [49,50]. However, the yield point phenomenon disappears as the sample size is reduced to the mesoscale; namely, the mesoscale specimen yields smoothly, leading to reduced YS. The yield point phenomenon is associated with low initial mobile dislocation density and the requirement of greater stresses to activate dislocation sources [49,50]. It is well known that the sample surface can also act as a dislocation source and enhance dislocation proliferation. Therefore, elevated stresses might be unnecessary to trigger the plastic yielding of surface grains. For the mesoscale specimens, there are only about 15 grains in the thickness direction. The surface grains are estimated to occupy a volume fraction of 20% and probably eliminate the yield point behavior. Such a scale dependence of the yield point phenomenon deserves further investigation.

### 3.4. Strain Hardening Behavior

In addition to strength and tensile ductility, strain hardening behavior is also of significant importance. The strain hardening can be represented by strain hardening exponent *n* in the Hollomon equation:

*σ* = *Kε^n^*, (1)

where *σ* and *ε* are true stress and true strain calculated from the engineering stress and engineering strain, and *K* is a constant. Table 2 lists the *n* values calculated from the uniform deformation stage (from about 3% to the initiation of necking) of the tensile curves for specimens at different length scales. The *n* values for the standard and sub-sized tensile specimens are 0.092 and 0.093, respectively, whereas for the mesoscale tensile specimen, the *n* value is lightly reduced to 0.085.

**Figure 8:**
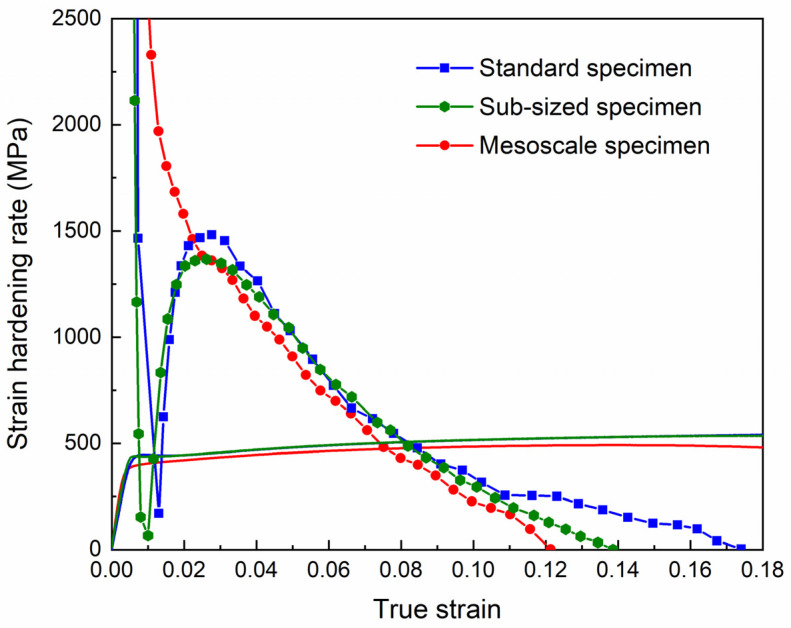
Variations of strain hardening rate (d*σ*/d*ε*) as a function of true strain (ε) for tensile tests of standard, sub-sized, and mesoscale specimens. The solid lines are the corresponding true stress–true strain curves.

The instantaneous strain hardening rate Θ = d*σ*/d*ε* as a function of true strain *ε* is plotted in Figure 8. The standard and sub-sized tensile specimens exhibit almost the same strain hardening behavior. Owing to the presence of the yield drop phenomenon, hardening for the standard and sub-sized tensile specimen is characterized by a sharp drop of Θ at a small strain followed by a rapid ascent to about 1500 MPa at a strain of approximately 0.03. At larger strains, Θ decreases monotonically with increasing strain. Different from that of the standard and the sub-sized specimen, Θ of the mesoscale tensile specimen rapidly drops in a monotonic manner at small strains due to the absence of the yield drop phenomenon. At strains exceeding 0.03, the trend of Θ reduction with strain is analogous to that of the standard and sub-sized specimens. However, the Θ values of the mesoscale specimen are always slightly smaller, resulting in a bit reduction in uniform elongation (Table 2).

### 3.5. Fracture Morphologies

**Figure 9:**
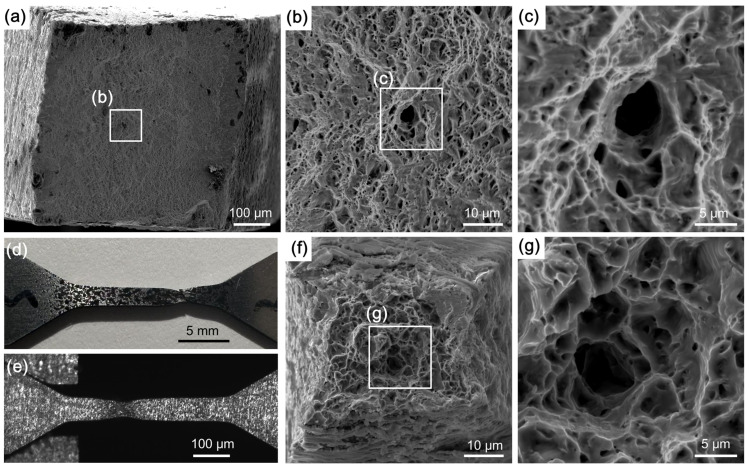
(**a**–**c**) Tensile fracture surface of the Zircaloy-4 alloy tested by sub-sized tensile specimen; (**d**) morphology of the sub-sized tensile specimen after tensile test; (**e**) morphology of the mesoscale tensile specimen close to fracture; (**f**,**g**) tensile fracture surface of the Zircaloy-4 alloy tested by mesoscale tensile specimen.

In order to investigate the fracture mode at different length scales, the fracture morphologies of the sub-sized and mesoscale tensile specimens were examined by SEM. Figure 9a displays the overview of the entire fracture surface of the sub-sized specimen, and Figure 9b shows the magnified observations on the center regions of the fracture surface. There are numerous equiaxed ductile dimples, suggesting that the fracture of the Zircaloy-4 alloy at room temperature is mediated by micro-void nucleation, growth, and coalescence. For comparison, Figure 9d,e present the sample morphologies of the sub-sized tensile specimen after fracture and of the mesoscale tensile specimen close to fracture. The development of localized necking is similar for both samples, irrespective of the large difference in sample dimensions. Figure 9f shows the overview of the entire fracture surface of the mesoscale tensile specimen, where substantial necking prior to the fracture is clearly visible. The fracture surface is also characterized by plenty of ductile dimples. Figure 9g displays the magnified observations on the center regions of the fracture surface. Closer examinations of the details of the dimples at high magnifications (Figure 9c,g) reveal that the dimple morphology and size are identical. This indicates that there is no distinctive variation in the fracture behavior and explains why close fracture elongations were measured at both length scales.

## 4. Discussion

The comparative tensile tests of the Zircaloy-4 alloy at different length scales demonstrate that bulk mechanical properties, including strength, strain hardening, and ductility, can be accurately measured by mesoscale tensile tests. This conclusion is different from numerous previous studies that reveal significant sample size dependence of the mechanical properties [20,46,51,52,53]. The underlying reasons for such a difference are discussed in the following text.

The effect of sample size on mechanical properties, in general, can arise from various intrinsic or extrinsic factors. Among them, the most important is the ratio of minimum sample dimension (thickness or diameter) to grain size (*t*/*d*). When *t*/*d* is smaller than some critical value, generally 5–10 for most materials [46,54,55], the surface grains that deform under weaker constraints of neighbor grains take precedence. Consequently, the flow strength, strain hardening capacity, and tensile ductility are impaired [46,56]. When *t*/*d* is greater than the critical value, the strength becomes sample-size-independent, but the ductility is still contingent upon the gauge geometry. In general, the elongation to failure decreases with increasing slimness ratio *S* = *LA*^−1/2^ and aspect ratio *R* = *w*/*t*, where *A* = *wt* is gauge section area [51,57]. Therefore, it is a standard practice to keep constant *S* and *R* values for the purpose of obtaining comparable ductility; however, this rule is frequently unmet for numerous small-scale mechanical tests [58]. In addition to microstructure and specimen geometry, the reliability and accuracy of the mechanical testing apparatus itself can also affect the results. In this regard, the accuracy of strain measurement is of utmost importance, and it determines whether comparable ductility can be obtained by small-scale mechanical tests. The small-scale mechanical tests frequently employ cross-head displacement to determine imposed strain [29,59]. However, the cross-head displacement inevitably includes the deformation outside the gauge section, leading to artificially greater ductility [60]. Lastly, for materials with high strain rate sensitivity, the strain rate can also influence the mechanical properties if different strain rates are used at different length scales.

This study aims to demonstrate that bulk mechanical properties can be accurately assessed by mesoscale tensile tests. Therefore, all the aforementioned factors have been delicately optimized to reduce their influences on the results. The gauge dimensions are proportionally scaled down from those of the standard specimens. Therefore, both *S* and *R* values are constant at different length scales. This guarantees that comparable ductility can be obtained. Moreover, to accurately measure the ductility, a contactless video gauging system (Figure 3), rather than cross-head displacement, was utilized to determine the deformation within the gauge (Figure 3b). The strain measurements at different length scales were implemented using the same DIC calculation algorithm developed in our lab, except that different optic magnifications were selected. Therefore, the ductility measurements at different length scales have almost the same precision, as shown in the standard deviations of the uniform and failure elongations in Table 2. In order to eliminate the influence of strain rate, the tensile tests were performed with almost the same strain rate. To verify this point, the real strain rates were calculated using the recorded strain and time. As summarized in Table 2, the strain rate of the mesoscale tensile tests (1.0 × 10^−3^ s^−1^) is very close to that of the standard and sub-sized tensile tests (9.0 × 10^−4^ s^−1^). However, as shown in Figure 4, the mesoscale tensile specimen contains only approximately 15 grains (*d* = 4.0 μm) in the thickness direction (*t* = 60 μm), and the volume fraction of surface grains is approximately 20%. The surface grains can deform more freely owing to the presence of more dislocation sources at the surface and to the weaker constraints from neighboring grains. As a result, the yield point phenomenon is completely suppressed, and the strain hardening is slightly reduced, leading to minor deviations in the measured mechanical properties (see Table 2 and Table 3). Augmenting the sample size can further increase the accuracy.

The comparatively reliable mesoscale tensile tests exhibit promising applications in various scientific and engineering fields when the sample volume available for mechanical tests is limited. For instance, small-scale mechanical testing has been in use in nuclear materials for decades [2,8] but is still constrained to sub-sized samples at the millimeter scale [20]. The extension to the mesoscale can save even more experimental space in the nuclear reactors and, more significantly, reduce the sample radioactivity levels. Furthermore, ion-beam irradiations have been frequently applied to explore material mechanical responses subjected to high-dose irradiation. But the ion-beam-irradiated layer is limited to a surface layer generally shallower than 100 μm. The mesoscale tensile tests can be directly employed to evaluate irradiation strengthening and embrittlement of such ion-beam-irradiated samples. In addition, the mesoscale tensile tests are also advantageous for monitoring the mechanical property degradation of structural components in service [14]. Because of the small sample volumes, the sample extraction can be almost nondestructive, and more samples can be extracted during the entire service life. Over the past years, high-throughput material synthesis and small-scale mechanical tests have attracted growing attention because of their promise to accelerate the material development cycle [6,61]. The combination of femtosecond laser micromachining and mesoscale tensile tests can greatly increase the efficiency of database development [61]. For all these potential applications, the high accuracy and almost size independence of the mechanical properties obtained by mesoscale tensile tests are of enormous advantages.

**Figure 10:**
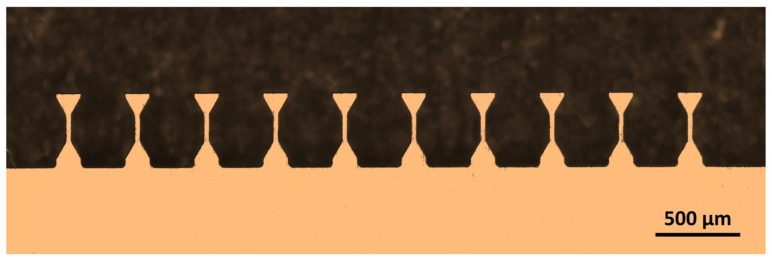
The micrograph shows 10 mesoscale tensile specimens with a gauge length of 250 μm and a gauge width of 60 μm from a metal sheet of 50 μm thickness.

It should be highlighted that the femtosecond laser indeed provides an efficient and precise tool for preparing high-quality specimens for mesoscale mechanical characterizations, as demonstrated in this study. The micrograph in Figure 10 again emphasizes the high efficiency of machining small-scale tensile specimens by femtosecond laser ablation. Ten tensile specimens with a gauge length of 250 μm, a gauge width of 60 μm, and a total length of 400 μm can be machined within 20 to 60 min from thin sheets of about 50 μm for most metals. This speed is, in fact, close to that of preparing macroscopic standard testing specimens via electro-discharging machining. Therefore, the mechanical characterizations at the mesoscale can be performed almost as efficiently as standard mechanical tests while holding the substantial advantages of small-scale mechanical characterizations. However, it should also be mentioned that the mechanical characterization based on femtosecond laser micro-machining is still limited by the current high costs of the femtosecond laser unit and high-precision motion stages required to prepare high-quality specimens. Meanwhile, potential applications to other, more complex materials have yet to be explored.

## 5. Conclusions

The mechanical properties of Zircaloy-4 alloy characterized by mesoscale tensile specimens fabricated by femtosecond laser ablation have been compared against those of standard and sub-sized specimens. The specimen geometry and experimental instruments are well-designed and optimized to reduce their potential influences on the obtained mechanical properties. Conclusions are summarized as follows:

The mechanical properties, including both strength and tensile ductility, can be accurately characterized by the mesoscale tensile tests. The quantitative results show that the YS and UTS values of mesoscale specimens are only −10.4% and −5.2% smaller than those of standard specimens, respectively. The UE and FE values at the mesoscale also deviate slightly by −12.5% and −13.5% from those of the standard specimens, respectively.

For a Zr alloy with a grain size of 4 μm, when the specimen thickness is reduced to ~60 μm, the surface grains occupy a volume fraction of ~20%. The large volume fraction of the surface grains with more surface dislocation sources and accumulating fewer dislocations during deformation leads to the disappearance of the yield point phenomenon and slightly reduced strain hardening capacity at the mesoscale. Augmenting the sample size can further increase the accuracy.

## Figures and Tables

**Table 1:** Chemical composition (weight percent) of the Zircaloy-4 alloy.

Alloy	Sn	Fe	Cr	C	Si	Zr
Zircaloy-4	1.32	0.18	0.096	0.013	0.008	Balanced

**Table 2:** Mechanical properties of Zircaloy-4 alloy tested with standard, sub-sized, and mesoscale specimens. SR, strain rate; YS, 0.2% offset yield strength; UTS, ultimate tensile strength; UE, uniform elongation; FE, fracture elongation; *n*, strain hardening exponent.

Sample	SR (s^−1^)	YS (MPa)	UTS (MPa)	UE (%)	FE (%)	*n*
Standard	9.1 × 10^−4^	442 ± 3	464 ± 1	8.8 ± 0.3	31.0 ± 0.6	0.092
Sub-sized	9.0 × 10^−4^	428 ± 2	458 ± 2	8.9 ± 0.4	28.5 ± 2.9	0.093
Mesoscale	1.0 × 10^−3^	396 ± 13	440 ± 15	7.7 ± 0.5	26.8 ± 1.9	0.085

**Table 3:** Statistics analysis of mechanical properties acquired by sub-sized and mesoscale tensile specimens with respect to those by standard specimens.

Sample	Parameter	YS (MPa)	UTS (MPa)	UE (%)	FE (%)
Sub-sized	t statistic	2.018	3.801	−0.737	1.985
	*p*-value	0.115	0.054	0.503	0.128
	Accuracy (%)	−3.2	−1.3	1.1	−8
Mesoscale	t statistic	6.657	12.118	2.871	4.699
	*p*-value	0.001	0.0002	0.028	0.005
	Accuracy (%)	−10.4	−5.2	−12.5	−13.5

## Data Availability

The original contributions presented in this study are included in the article.
